# Association between major cardiovascular events and abiraterone acetate compared to enzalutamide in patients with metastatic castration-resistant prostate cancer: a post hoc analysis of the EVADE study

**DOI:** 10.1007/s00345-025-05841-9

**Published:** 2025-08-01

**Authors:** Amit Bahl, Andrew Chilelli, Rita Faria, Nigel Rozario, Robert Snijder, Sari Stark, Axel S. Merseburger

**Affiliations:** 1 Bristol Haematology and Oncology Centre, University Hospitals Bristol & Weston NHS Foundation, Bristol, UK; 2 Astellas Pharma Europe Ltd, Addlestone, Surrey, UK; 3 University Hospital Schleswig-Holstein, Campus Lübeck, Ratzeburger Allee, Lübeck, Germany

**Keywords:** Abiraterone acetate, Enzalutamide, Metastatic castration-resistant prostate cancer, Cardiovascular, Major cardiovascular events, EVADE study

## Abstract

**Purpose:**

This study investigated the risk of major cardiovascular (CV) events in patients with metastatic castration-resistant prostate cancer (mCRPC) treated with abiraterone acetate (AA) versus enzalutamide (ENZA); assessed treatments for prostate cancer (PC), focusing on corticosteroid-containing regimens; and examined comorbidities recorded in primary and secondary care.

**Methods:**

This was a post hoc analysis of the retrospective, observational EVADE study in patients who received AA or ENZA for mCRPC. Patient characteristics at treatment initiation were described, focusing on characteristics affecting the risk of CV events, together with PC treatments in the entire PC pathway. Major CV event risk was assessed with Cox regression after adjustment with inverse probability of treatment weighting (IPTW).

**Results:**

Overall, 1,382 patients were included (AA: 556; ENZA: 826). After IPTW adjustment, risk of a major CV event was 65.0% higher in the AA versus the ENZA group (hazard ratio: 1.65; 95% confidence interval: 1.27, 2.14; *P =* 0.0001). Across the entire patient pathway, the average time on corticosteroid-containing PC regimens was 298 days versus 72 days for the AA versus ENZA group. While type 2 diabetes was less frequently reported in secondary versus primary care (59.8% vs. 94.9%), CV comorbidities were more frequently reported (87.1% vs. 84.2%).

**Conclusion:**

Major CV event risk was significantly higher in the AA versus ENZA group, and the AA group had numerically greater exposure to corticosteroid-containing PC regimens over the treatment course. There were discrepancies in recording diabetes and CV comorbidities between primary and secondary care in England.

**Supplementary Information:**

The online version contains supplementary material available at 10.1007/s00345-025-05841-9.

## Introduction

The hormone therapies abiraterone acetate (AA) and enzalutamide (ENZA) are indicated for treatment of metastatic castration-resistant prostate cancer (mCRPC) [1, 2] and recommended in UK treatment guidelines [3–6]. There are no head-to-head clinical trials comparing ENZA to AA; however, real-world studies have shown better outcomes, including improved overall survival, for patients treated with ENZA versus AA [7–9].

Risk of cardiovascular (CV) events has been investigated by several studies, although none in the UK. These generally found that AA treatment was associated with increased risks of cardiac disorders [10–19].

However, the extent to which CV toxicity is due to AA or coadministration of corticosteroids is unclear, as cardiotoxicity has also been associated with cumulative corticosteroid use [20, 21].

Given the gaps in the evidence base, this post hoc analysis of the EVADE study aimed to: (i) investigate the risk of major CV events in patients with mCRPC treated with AA versus ENZA; (ii) assess treatments for PC prescribed to patients throughout their treatment pathway, with a particular focus on exposure to corticosteroid-containing regimens; (iii) and examine the recording of comorbidities in primary and secondary care. The EVADE study was a retrospective observational study in England, which found that mCRPC patients receiving AA were more likely to experience new onset or worsening type 2 diabetes mellitus (T2DM) when compared with patients receiving treatment with ENZA [22].

## Methods

### Data sources

The EVADE study used a sample of electronic medical records from the Clinical Practice Research Datalink GOLD (UK) and Aurum (England) databases, linked to the Hospital Episode Statistics, Public Health England Cancer Registry, and Systemic Anti-Cancer Treatment data sets [22]. EVADE included patients diagnosed with PC and treated with AA or ENZA between April 2015 and October 2021. Patients were assumed to be treated for mCRPC, given it was the only licensed and reimbursed indication for AA and ENZA during most of the study period [3–6]. Patients receiving AA, docetaxel, or cabazitaxel were assumed to receive concomitant steroids, as per licensed posology, with these treatments classified within the analysis as corticosteroid-containing regimens and time on treatment assumed to correspond to duration of exposure to corticosteroids [2, 23, 24]. For the comparison of major CV event risk between AA and ENZA groups, the period included time from first administration date of AA or ENZA (index date) to patient’s last observed data date/exit date from the study.

### Statistical analyses

The study period was defined as the first observed patient index date (January 2014) until the patients’ last observed data date (January 2022). For descriptive analyses, patient characteristics (e.g., comorbidities, demographics [including age, ethnicity and smoking status]) and outcomes (e.g., exposure to PC treatments) derived from the full patient history were described with summary statistics. Major CV events were defined as an inpatient admission or the primary cause of death for which the primary diagnosis was atherosclerosis, arrhythmia, atrioventricular block, cardiac arrest, cerebrovascular disease, deep vein thrombosis (DVT), heart failure, hypertension, ischemic heart disease, nonrheumatic valve disorders, peripheral vascular disease, and pulmonary circulation disorders. Characteristics included in the analysis were based on those investigated in a real-world study comparing the CV event risk between AA- and ENZA-treated patients [19], complemented by major CV risk factors included in the UK QRISK3 risk prediction model [25]. Differences in patient characteristics between treatment groups were assessed using standardized mean difference (SMD). SMD < 0.1 (absolute value) was considered unlikely to be clinically meaningful. *P*‑values (statistically significant at the 5% level) were calculated using chi-square tests for categorical values and t-tests for continuous variables. Differences were considered to be noteworthy if SMD > 0.1 or *P* < 0.05. Inverse probability of treatment weighting (IPTW) was used to reduce observed differences between treatment groups and improve their comparability. IPTW adjustments included these patient characteristics at index date: age, ethnicity, CV disease (CVD) history and comorbidities of interest, procedures of interest, and medications (Online Resource 1). Cox regression was used to estimate the hazard ratio (HR) for major CV events, pre- and post-IPTW adjustment.

To assess treatments for PC, Sankey plots were generated to describe PC treatments preceding and following AA or ENZA treatment initiation, based on the full patient history.

To examine the recording of comorbidities, counts and percentages of patients with comorbidities recorded in primary (general practice) care data, secondary (hospital) care data, either primary or secondary care data, or in both primary and secondary care data were tabulated.

## Results

### Study population

A total of 1,382 patients were included (AA: 556; ENZA: 826). The EVADE study population was predominantly white (89.0%), with a mean (standard deviation [SD]) age of 72.7 (9.1) years at initiation of AA or ENZA treatment (Table 1). Patients on ENZA had longer median (IQR) durations of treatment (144 [83.0–285.0] days) than those on AA (121.5 [58.0–266.0] days).

**Table 1:** Demographic characteristics at index date and comorbidities prior to the index date in patients with mCRPC in the EVADE study

	Statistic	AA (*n* = 556)	ENZA (*n* = 826)	Total (*N* = 1382)	SMD^a^	*P*-value
Age at index (years)	–	–	–	–	–	0.0794
	Mean (SD)	73.2 (9.08)	72.4 (9.07)	72.7 (9.08)	0.088	–
	Median (IQR)	74.0 (67.0–80.0)	72.0 (67.0–79.0)	73.0 (67.0–79.0)	–	–
	Range	47–96	43–95	43–96	–	–
Ethnicity^b^	–	–	–	–	–	0.3127
White	n (%)	504 (90.6)	726 (87.9)	1230 (89.0)	0.087	–
Black/African/ Caribbean/Black British	n (%)	24 (4.3)	38 (4.6)	62 (4.5)	−0.015	–
Other ethnic group	n (%)	16 (2.9)	30 (3.6)	46 (3.3)	−0.039	–
Asian/Asian British	n (%)	7 (1.3)	17 (2.1)	24 (1.7)	−0.062	–
Mixed/Multiple ethnic groups	n (%)	4 (0.7)	6 (0.7)	10 (0.7)	0	–
Missing	n (%)	1 (0.2)	9 (1.1)	10 (0.7)	−0.112	–
Smoking status	–	–	–	–	–	0.6477
Never smoker	n (%)	307 (55.2)	429 (51.9)	736 (53.3)	0.066	–
Ex-smoker	n (%)	145 (26.1)	227 (27.5)	372 (26.9)	−0.032	–
Current smoker	n (%)	78 (14.0)	131 (15.9)	209 (15.1)	−0.053	–
Missing	n (%)	26 (4.7)	39 (4.7)	65 (4.7)	0	–
CCI score	–	–	–	–	–	0.7668
	n	556	826	1382	–	–
	Mean (SD)	11.3 (3.32)	11.3 (3.51)	11.3 (3.43)	0	–
	Median (IQR)	12.0 (10.0–13.0)	11.0 (10.0–13.0)	12.0 (10.0–13.0)	–	–
	Range	2–21	2–21	2–21	–	–
CHA_2_DS_2_-VASc^c^ score	–	–	–	–	–	0.5634
	n	556	826	1382	–	–
	Mean (SD)	2.2 (1.34)	2.2 (1.41)	2.2 (1.38)	0	–
	Median (IQR)	2.0 (1.0–3.0)	2.0 (1.0–3.0)	2.0 (1.0–3.0)	–	–
	Range	0–7	0–8	0–8	–	–
Pre-index CVD history						
Any CVD	n (%)	379 (68.2)	574 (69.5)	953 (69.0)	−0.028	0.6013
Any CVD excluding cerebrovascular disease	n (%)	373 (67.1)	571 (69.1)	944 (68.3)	−0.043	0.4237
Hypertension	n (%)	304 (54.7)	477 (57.7)	781 (56.5)	−0.060	0.2586
Ischemic heart disease	n (%)	100 (18.0)	167 (20.2)	267 (19.3)	−0.056	0.3027
Arrhythmia	n (%)	95 (17.1)	133 (16.1)	228 (16.5)	0.027	0.6287
Cerebrovascular disease	n (%)	61 (11.0)	64 (7.7)	125 (9.0)	0.114	0.0405
Peripheral vascular disease	n (%)	40 (7.2)	64 (7.7)	104 (7.5)	−0.019	0.7019
Congestive heart failure	n (%)	24 (4.3)	59 (7.1)	83 (6.0)	−0.121	0.0301
Deep vein thrombosis	n (%)	19 (3.4)	55 (6.7)	74 (5.4)	−0.151	0.0087
Pulmonary circulation disorders	n (%)	26 (4.7)	46 (5.6)	72 (5.2)	−0.041	0.4640
Nonrheumatic valve disorders	n (%)	26 (4.7)	42 (5.1)	68 (4.9)	−0.019	0.7306
Atrioventricular block	n (%)	22 (4.0)	37 (4.5)	59 (4.3)	−0.025	0.6375
Atherosclerosis	n (%)	8 (1.4)	9 (1.1)	17 (1.2)	0.027	0.5635
Cardiac arrest	n (%)	0 (0.0)	4 (0.5)	4 (0.3)	−0.1	0.1003
Pre-index T2DM^d^						
T2DM	n (%)	116 (20.9)	217 (26.3)	333 (24.1)	−0.127	0.0212
T2DM and any CVD	n (%)	95 (17.1)	182 (22.0)	277 (20.0)	−0.124	0.0243
T2DM with complications	n (%)	69 (12.4)	144 (17.4)	213 (15.4)	−0.141	0.0112
T2DM with complications unspecified	n (%)	47 (8.5)	73 (8.8)	120 (8.7)	−0.011	0.8034
T2DM without complications	n (%)	4 (0.7)	7 (0.8)	11 (0.8)	−0.012	0.7928
Pre-index other comorbidities of interest						
Hyperlipidemia	n (%)	204 (36.7)	301 (36.4)	505 (36.5)	0.006	0.9246
Urinary tract infection	n (%)	107 (19.2)	158 (19.1)	265 (19.2)	0.003	0.9571
Renal impairment	n (%)	103 (18.5)	149 (18.0)	252 (18.2)	0.013	0.8184
Obesity	n (%)	57 (10.3)	110 (13.3)	167 (12.1)	−0.093	0.0864
Anemia	n (%)	71 (12.8)	94 (11.4)	165 (11.9)	0.043	0.4346
COPD	n (%)	53 (9.5)	84 (10.2)	137 (9.9)	−0.023	0.6975
Liver damage or abnormality	n (%)	50 (9.0)	79 (9.6)	129 (9.3)	−0.021	0.7203
Malignant neoplasm of the skin	n (%)	6 (1.1)	11 (1.3)	17 (1.2)	−0.018	0.6761
Cardiomyopathy	n (%)	5 (0.9)	11 (1.3)	16 (1.2)	−0.038	0.4612
SLE	n (%)	0 (0.0)	0 (0.0)	0 (0.0)	NC	NC
Index Year	–	–	–	–	–	< 0.0001
2014	n (%)	7 (1.3)	4 (0.5)	11 (0.8)	0.085	–
2015	n (%)	41 (7.4)	67 (8.1)	108 (7.8)	−0.026	–
2016	n (%)	86 (15.5)	213 (25.8)	299 (21.6)	−0.257	–
2017	n (%)	170 (30.6)	266 (32.2)	436 (31.5)	−0.034	–
2018	n (%)	150 (27.0)	148 (17.9)	298 (21.6)	0.219	–
2019	n (%)	75 (13.5)	86 (10.4)	161 (11.6)	0.096	–
2020	n (%)	27 (4.9)	38 (4.6)	65 (4.7)	0.014	–
2021	n (%)	0 (0)	4 (0.5)	4 (0.3)	−0.100	–

*AA* abiraterone acetate, *ADT* androgen-depravation therapy, *CCI* Charlson Comorbidity Index, *COPD* chronic obstructive pulmonary disease, *ENZA* enzalutamide, *IQR* interquartile range, *mCRPC* metastatic castration-resistant prostate cancer, *NC* not calculated, *SMD* standardized mean difference, *T2DM* type 2 diabetes mellitus.

^a^SMD was used to assess differences between the AA and ENZA index treatment groups (SMD < 0.1 [absolute value] was considered unlikely to be clinically meaningful). A dash (–) means that the SMD calculation was not applicable for that row. *P*-value was provided for reference (differences were statistically significant at the 5% level).

^b^Ethnicity data were obtained any time prior to index date.

^c^CHA_2_DS_2_-VASc is defined as congestive heart failure; hypertension; age ≥ 75 years (doubled); diabetes mellitus, prior stroke or transient ischemic attack or thromboembolism (doubled); vascular disease; age 65 to 74 years; female.

^d^Patients may have been classified in more than one T2DM category; therefore, the number of patients with T2DM may not be equal to the total number of patients in the categories of T2DM with complications, T2DM without complications, and T2DM complications unspecified.

### Prior comorbidities

Approximately two-thirds (69.0%) of patients had any CVD prior to index date (Table 1). One-fifth of patients had both T2DM and CV comorbidities. Prior comorbidities were generally well balanced between treatment groups, with the exception of history of T2DM and CVD: a larger percentage of patients in the ENZA group had T2DM (26.3% vs. 20.9%; SMD = − 0.127; *P* = 0.0212), as well as T2DM with complications (17.4% vs. 12.4%; SMD = − 0.141; *P* = 0.0112), congestive heart failure (7.1% vs. 4.3%; SMD = − 0.121; *P* = 0.0301), and DVT (6.7% vs. 3.4%; SMD = − 0.151; *P* = 0.0087). A larger proportion of patients in the AA group had cerebrovascular disease (11.0% vs. 7.7%; SMD = 0.114; *P* = 0.0405).

### Prior medications and procedures

Medication use prior to index date was similar between AA and ENZA treatment groups; however, a larger proportion of patients subsequently treated with ENZA had previously been prescribed first-generation anti-androgens (bicalutamide, flutamide, nilutamide, and cyproterone acetate) versus patients treated with AA (66.0% vs. 59.0%; SMD = − 0.145; *P =* 0.0083). Prostatectomy was the most common pre-index procedure of interest (41.2%). There was a notable difference in the proportion of patients who were reported to have had orchiectomy (ENZA: 0.6%, AA: 2.7%; SMD = 0.165; *P* = 0.0014), but numbers were small (*n* = 20).

### Incidence of major CV events

Prior to IPTW adjustment, covariates between treatment groups were similar based on SMD values, except for T2DM, T2DM with complications, first-generation anti-androgens, cerebrovascular disease, DVT, congestive heart failure, and orchiectomy. After IPTW adjustment, all covariates, including T2DM and CVD history, were similar (SMD < 0.1) (Online Resource 1).

**Fig. 1:**
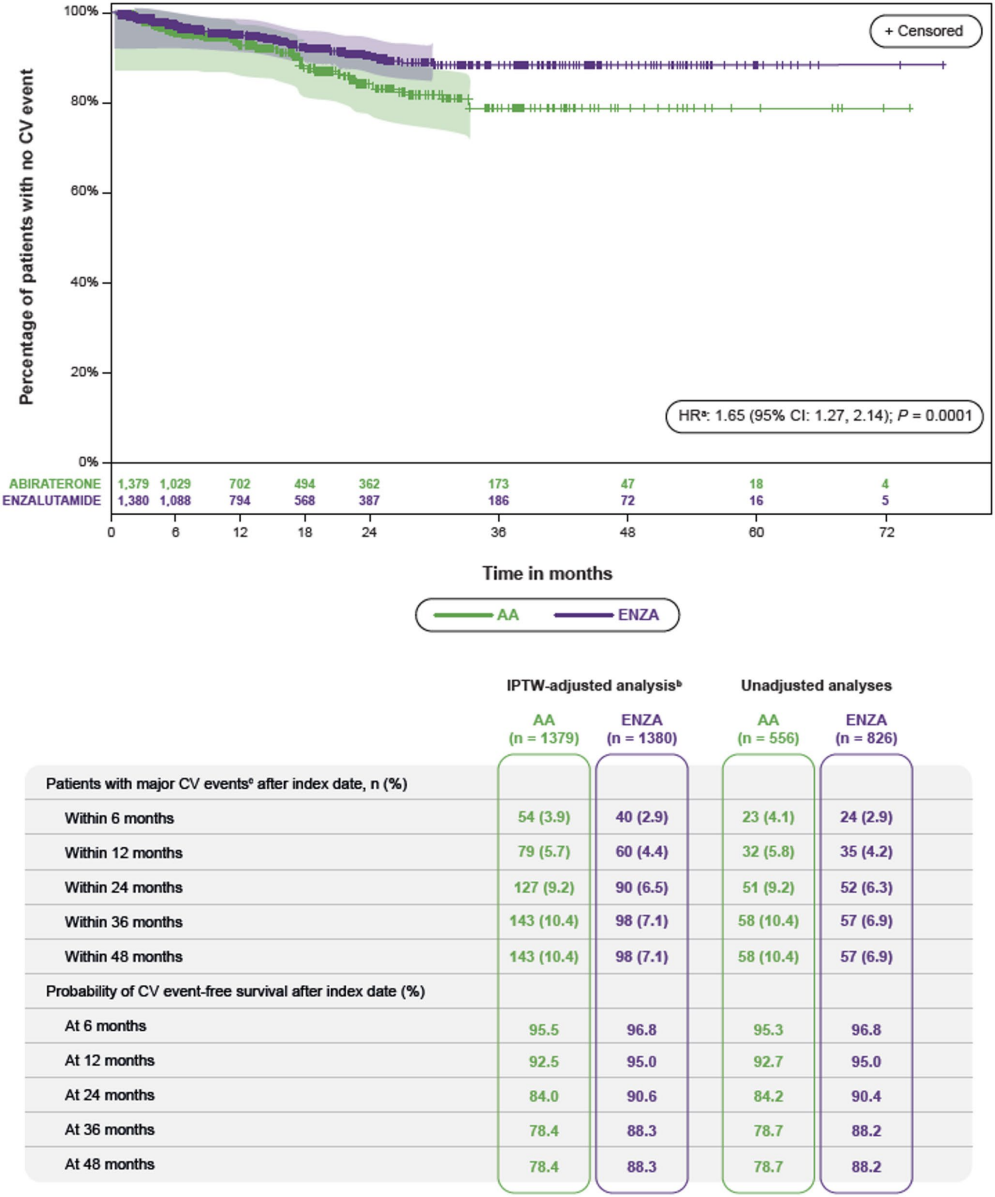
Incidence of major CV events over time following treatment initiation (IPTW-adjusted). *AA* abiraterone acetate, *CI* confidence interval, *CV* cardiovascular, *ENZA* enzalutamide, *HR* hazard ratio, *IPTW* inverted probability of treatment weighting, *mCRPC* metastatic castration-resistant prostate cancer. Patients who died from other causes than a major CV event were censored. Shaded areas represent 95% CIs of the proportion of patients without an event. The number of events observed ended earlier in the ENZA vs. AA treatment group; therefore, the shaded areas representing the 95% CIs ended earlier for the ENZA vs. AA treatment group. ^a^Comparison of time to CV event via Cox proportional hazard model; enzalutamide = comparator; IPTW adjustment with 95% Hall-Wellner bands is shown. ^b^The numbers of patients in the IPTW-adjusted analyses are artificially inflated with the IPTW procedure, because patients are upweighted so that two treatment groups with similar characteristics are compared. ^c^Major CV events, defined as a primary diagnosis for an inpatient admission or the primary cause of death, included atherosclerosis, arrhythmia, atrioventricular block, cardiac arrest, cerebrovascular disease, deep vein thrombosis, heart failure, hypertension, ischemic heart disease, nonrheumatic valve disorders, peripheral vascular disease, and pulmonary circulation disorders

In the non–IPTW-adjusted analysis, major CV events were observed in 8.3% (115/1382) of all patients after AA or ENZA initiation, higher in AA- versus ENZA-treated patients (10.4% [58/556] vs. 6.9% [57/826]). After IPTW adjustment, AA-treated patients had a 65.0% higher risk of major CV events versus the ENZA-treated group (HR: 1.65; 95% CI: 1.27, 2.14; *P =* 0.0001) (Fig. 1). Median and 25th percentile for the time to a major CV event were not reached. The probability of CV event-free survival at 48 months for AA- versus ENZA-treated patients was 78.4% versus 88.3% (Fig. 1). The number of patients needed to treat with ENZA versus AA to prevent one major CV event was 64 patients at 6 months, 47 patients at 12 months, 25 patients at 24 months, and 21 patients at 36 and 48 months.

### Assessment of treatments and exposure to corticosteroid-containing PC regimens

The most common (> 10%) medications prescribed prior to ENZA or AA treatment were first-generation anti-androgens (63.2%), opioids (51.9%), androgen-deprivation therapy (ADT; 48.2%), antiplatelet therapy (36.9%), and anticoagulants (17.4%). ADT alone was the most prescribed PC medication after ENZA or AA treatment. Before ENZA or AA treatment, 35.8% of patients who subsequently received AA and 32.7% who subsequently received ENZA had received corticosteroid-containing PC regimens (average time on steroid-containing regimen: AA, 150 days; ENZA, 141 days; Online Resource 2). During treatment with ENZA, 5.6% of patients received corticosteroid-containing PC regimens for a mean duration of 15 days. Given that AA is commonly administered with corticosteroids, the exposure to corticosteroids during AA treatment corresponds to the AA treatment duration, which was 218 days, on average. After AA or ENZA treatment, 19.2% of patients treated with AA and 16.9% treated with ENZA received a subsequent corticosteroid-containing PC regimen (average time on corticosteroid-containing PC regimen: AA, 135 days; ENZA, 148 days). Across the entire PC patient pathway following diagnosis, the average time on corticosteroid-containing regimens was 298 days versus 164 days for patients treated with AA and ENZA, respectively (Online Resource 2). When accounting for the total population (i.e., including those who did and did not receive corticosteroids), the total exposure to corticosteroids was 298 days for AA-treated patients and 72 days for ENZA-treated patients (Online Resource 3).

### Comorbidity recording in primary or secondary care

The proportion of patients with T2DM was under-reported in secondary care (59.8%) versus primary care (94.9%; Online Resource 4). Conversely, a higher proportion of patients with CV comorbidities were recorded in secondary (87.1%) versus primary care (84.2%). A similar pattern was observed in the AA and ENZA treatment groups, with under-reporting of T2DM in secondary care and of CV comorbidities in primary care (Online Resource 4).

## Discussion

The risk of major CV events was greater in patients who received AA than in those who received ENZA. To our knowledge, this is the first UK study to determine the risk of experiencing major CV events in patients with mCRPC treated with AA or ENZA. This finding is aligned with meta-analyses, claims database analyses, and pharmacovigilance database studies, which found increased risk of CV events in patients treated with AA versus ENZA [11–19]. For example, the adjusted HR is consistent with the recent real-world study comparing AA with ENZA in Germany, which also found lower CV event rates in patients treated with ENZA versus AA (HR: 0.70, 95% CI: 0.57–0.86; present study HR: 0.61; 95% CI: 0.47–0.78) [19].

AA-treated patients had numerically longer exposure to corticosteroid-containing PC regimens than ENZA-treated patients (298 vs. 72 days). This longer exposure was mostly driven by the period of treatment with AA, given that concomitant use of corticosteroid is needed to compensate for reductions in serum cortisol and to block the compensatory increase in adrenocorticotropic hormone [22, 26]. As corticosteroids have been associated with cardiotoxicity [20, 21] and higher corticosteroid doses with higher risk [27], the greater cumulative corticosteroid use in the AA-treated patients could underlie observed differences in the risk of CV events. An area of future research is whether larger differences in CV event risk would be found in patients with mHSPC, given the longer AA exposure.

A pattern emerged of under-recording of T2DM in secondary versus primary care and of CV comorbidities in primary versus secondary care. These findings suggest that comorbidities are not comprehensively recorded in either primary or secondary care, which may lead to an underestimation of comorbidity prevalence when using these data for real-world studies, with potential risk of bias. In this setting, the use of a primary care database linked to secondary care, when available, is likely to provide more comprehensive estimates of comorbidities in patients with mCRPC. Moreover, secondary care databases may provide more comprehensive estimates of CV comorbidity prevalence than primary care databases.

The differences in patients’ history at baseline may influence physician preferences in prescribing ENZA over AA and vice versa. For example, the preference for ENZA in patients with pre-existing diabetes given the higher risks of diabetes and hyperglycaemia with AA [22, 28, 29]; whereas, AA may be preferred in patients with prior cerebrovascular disease because seizures have been reported in 0.6% of patients on ENZA [1]. Similar differences in prescribing patterns were also observed in recent UK and German studies [19, 30], supporting that there are differences in how AA and ENZA are used in clinical practice.

This study had certain limitations. The comparison of T2DM and CVD prevalence between primary and secondary care records was qualitative rather than formally statistical and assumed that comorbidities were present if recorded; however, comorbidities may have been recorded in error, or not recorded in either database, despite diagnosis. Additionally, testosterone levels were not available in either database. It was assumed that treatments were accurately recorded and used according to their licensed indications, including that treatments licensed in combination with steroids (i.e., AA, cabazitaxel, docetaxel) were always prescribed with concomitant steroids; however, recording errors and/or off-license prescribing, or adjustments to steroid co-administration may have occurred. Therefore, exposure to steroids may be different in practice. Calculations of treatment duration may have underestimated actual duration due to the assumption that the date of last treatment corresponded to the date when patients were last observed, while patients may have continued treatment after the period of data availability. Pre-index ADT use was lower than expected given that AA and ENZA were licensed only for mCRPC for most of the study period, therefore some instances of under-recording/missing data may have occurred. However, as the proportion of ADT use recorded in the database was similar between treatment groups, and there is no reason to believe that actual ADT use would be different prior to subsequent ENZA or AA treatment, the impact of this apparent under-recording on major CV event risk is expected to be minor. Although confounding bias could have affected the comparison of CV risk associated with use of AA versus ENZA, the methodology aimed to minimize this by using a lookback period as far as the data allowed for patients’ clinical history and including a large range of characteristics that may affect CV risk as adjustment factors. Causation should not be directly inferred from this study alone, as this was a post hoc analysis of an observational study; rather, causation should be interpreted in the context of other studies comparing AA to ENZA and of previous literature on the risk of corticosteroids, which are used concomitantly with AA.

## Conclusion

Consistent with previous studies in other countries, the risk of major CV events was significantly higher in patients receiving AA versus ENZA for mCRPC in England. AA-treated patients had numerically longer exposure to corticosteroid-containing PC regimens than ENZA-treated patients throughout their PC treatment pathway, primarily due to concomitant use of corticosteroids with AA. There are discrepancies in the recording of T2DM and CV comorbidities between primary and secondary care in England, which may have the potential to impact real-world studies.

## Supplementary Information

Below is the link to the electronic supplementary material.

Supplementary Material 1

## Data Availability

Details for how researchers may request access to anonymized participant level data, trial level data, and protocols from Astellas sponsored clinical trials can be found at https://www.clinicaltrials.astellas.com/transparency/.
