# Supplementary material for: Association between major cardiovascular events and abiraterone acetate compared to enzalutamide in patients with metastatic castration-resistant prostate cancer: a post hoc analysis of the EVADE study
Source: World J Urol. 2025 Aug 1;43(1):465. doi: 10.1007/s00345-025-05841-9 (PMC12316759; doi:10.1007/s00345-025-05841-9)
Supplement: Supplementary file 1 — Supplementary Material 1 [file 345_2025_5841_MOESM1_ESM.docx]

## Online Resources

**Association between major cardiovascular events and abiraterone acetate compared to enzalutamide in patients with metastatic castration-resistant prostate cancer: a post hoc analysis of the EVADE study**

Amit Bahl,*^a^ Andrew Chilelli,^b^ Rita Faria^b^, Nigel Rozario,^b^ Robert Snijder,^b^ Sari Stark,^b^ Axel S. Merseburger^c^

^a^Bristol Haematology and Oncology Centre, University Hospitals Bristol & Weston NHS Foundation, Bristol, UK

^b^Astellas Pharma Europe Ltd, Addlestone, Surrey, UK

^c^University Hospital Schleswig-Holstein, Campus Lübeck, Ratzeburger Allee, Lübeck, Germany

*Corresponding author

### Online Resource 1.

#### **Assessment of propensity score weighting**

The covariates (potential confounders) included in the Cox regression model are described below. Standardized mean difference (SMD) values for each confounder are shown in the figure and table below followed by comparative density of propensity score curves for the abiraterone acetate (AA) and enzalutamide (ENZA) treatment groups.

After inverse probability of treatment weighting (IPTW) adjustment, SMDs across all baseline characteristics, including diabetes and cardiovascular (CV) disease history, were similar (i.e., SMD < 0.1) between the AA and ENZA treatment groups.

#### **SMD with and without propensity score weights**

| Variable | SMD before weighting | SMD after weighting |
| --- | --- | --- |
| Anemia | 0.043 | 0.012 |
| Arrhythmia | 0.026 | −0.021 |
| Atherosclerosis | 0.031 | −0.035 |
| Atrioventricular block | −0.026 | 0.010 |
| Brachytherapy/Radiotherapy | −0.010 | −0.041 |
| Cardiomyopathy | −0.041 | 0.037 |
| Cerebrovascular disease | 0.111 | 0.000 |
| Chronic obstructive pulmonary disease | −0.021 | 0.000 |
| Congestive heart failure | −0.122 | 0.008 |
| Deep vein thrombosis | −0.149 | −0.009 |
| Diabetes, type 2 | −0.128 | −0.014 |
| Diabetes unspecified | −0.014 | 0.000 |
| Diabetes with complications | −0.141 | −0.017 |
| Diabetes without complications | −0.015 | 0.000 |
| First-generation anti-androgens | −0.145 | −0.045 |
| Hyperlipidemia | 0.005 | −0.024 |
| Hypertension | −0.062 | −0.012 |
| Ischemic heart disease | −0.057 | −0.020 |
| Liver damage or abnormality | −0.020 | −0.034 |
| Malignant neoplasm of the skin | −0.023 | −0.018 |
| Nonrheumatic valve disorders | −0.019 | −0.009 |
| Obesity | −0.095 | −0.024 |
| Opioid history | 0.051 | 0.012 |
| Orchiectomy | 0.165 | 0.000 |
| Peripheral vascular disease | −0.021 | −0.007 |
| Prostatectomy pre−index | −0.020 | 0.000 |
| Renal impairment | 0.013 | 0.030 |
| Urinary tract infection | 0.003 | 0.035 |
| Use of anticoagulants | 0.056 | −0.005 |
| Use of antiplatelet drug or therapy | −0.039 | −0.024 |
| Use of drugs affecting bone structure and mineralization | 0.058 | 0.000 |
| Age | −0.096 | −0.003 |
| Ethnicity | 0.089 | 0.000 |

SMD, standardized mean difference.

#### **Assessment of propensity score weighting**


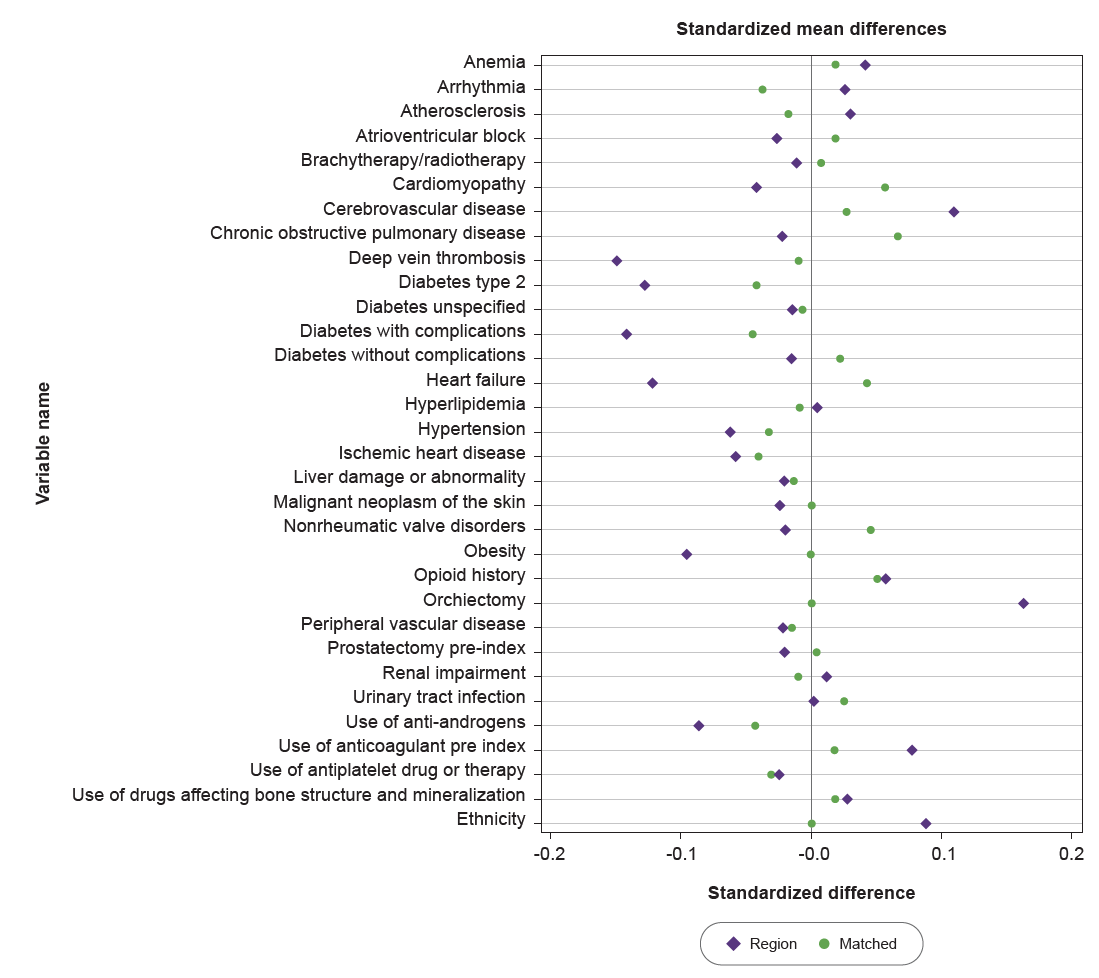


Region: prior to IPTW

#### **Comparative density of propensity scores showed overlap in the probability of being treated with AA or ENZA, except for a small proportion of patients with low propensity scores**


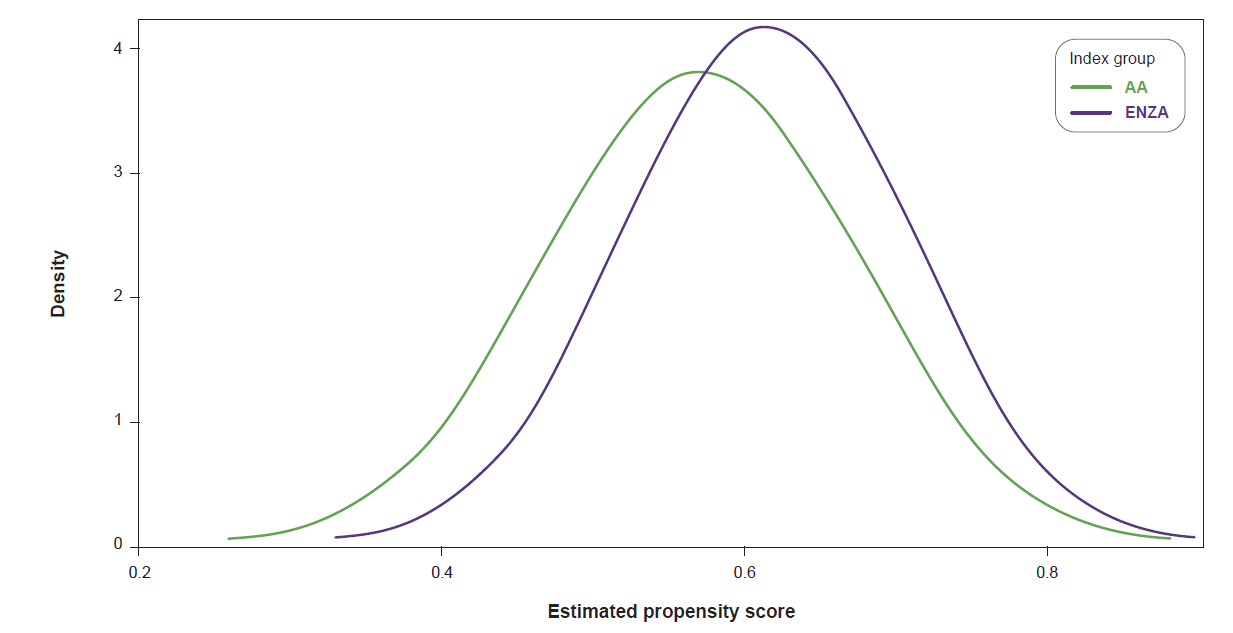


AA, abiraterone acetate; ENZA, enzalutamide

### Online Resource 2.

#### **Prostate cancer treatment duration before, during, and after AA or ENZA index treatment in patients with mCRPC in the EVADE study**

| Treatment/  Duration (days) | Statistic | AA (n = 556) | | | | ENZA (n = 826) | | | |
| --- | --- | --- | --- | --- | --- | --- | --- | --- | --- |
|  |  | Pre-index | During index | Post-index | Overall | Pre-index | During index | Post-index | Overall |
| Corticosteroid-containing regimens^a^ | n (%) | 199 (35.8) | 556 (100.0) | 107 (19.2) | 556 (100.0) | 270 (32.7) | 46 (5.6) | 140 (16.9) | 363 (43.9) |
|  | Median (IQR) | 134 (123–160) | 140  (82–281) | 116  (55–194) | 246 (127–394) | 134 (116–155) | 9 (3–12) | 134 (71–201) | 141 (127–197) |
|  | Mean (SD) | 150 (69.7) | 218 (217.8) | 135 (102.7) | 298 (233.4) | 141 (60.0) | 15 (27.5) | 148 (109.8) | 164 (88.5) |
| ADT alone^b^ | n (%) | 239 (43.0) | 200 (36.0) | 128 (23.0) | 263 (47.3) | 366 (44.3) | 275 (33.3) | 207 (25.1) | 411 (49.8) |
|  | Median (IQR) | 238 (85–558) | 85 (29–197) | 124 (54–273) | 394 (114–841) | 319 (112–563) | 108 (34–206) | 164 (49–375) | 452 (135–851) |
|  | Mean (SD) | 355 (361.0) | 149 (173.9) | 211 (243.3) | 538 (547.7) | 371 (301.2) | 154 (152.1) | 283 (312.1) | 576 (528.1) |
| Abiraterone | n (%) | . | 556 (100.0) | 16 (2.9) | 556 (100.0) | . | . | 25 (3.0) | 25 (3.0) |
|  | Median (IQR) | . | 139 (72–296) | 29 (7–130) | 140 (83–284) | . | . | 134 (29–176) | 134 (29–176) |
|  | Mean (SD) | . | 225 (236.6) | 105 (168.6) | 221 (220.8) | . | . | 166 (172.8) | 166 (172.8) |
| Cabazitaxel | n (%) | 17 (3.1) | 24 (4.3) | 56 (10.1) | 73 (13.1) | 15 (1.8) | 6 (0.7) | 56 (6.8) | 71 (8.6) |
|  | Median (IQR) | 126 (86–141) | 9 (1–21) | 99 (42–147) | 112 (51–142) | 110 (71–189) | 10 (8–12) | 92 (68–134) | 92 (71–136) |
|  | Mean (SD) | 120 (53.9) | 11 (10.9) | 113 (80.2) | 118 (72.8) | 122 (70.2) | 10 (2.9) | 102 (55.1) | 107 (59.0) |
| Carboplatin | n (%) | . | . | 7 (1.3) | 7 (1.3) | 9 (1.1) | 8 (1.0) | 8 (1.0) | 17 (2.1) |
|  | Median (IQR) | . | . | 76 (71–89) | 76 (71–112) | 126 (71–148) | 7 (2–22) | 61 (37–113) | 86 (51–140) |
|  | Mean (SD) | . | . | 94 (41.8) | 97 (42.1) | 117 (53.4) | 11 (11.8) | 79 (52.6) | 104 (60.2) |
| Docetaxel | n (%) | 199 (35.8) | 17 (3.1) | 52 (9.4) | 246 (44.2) | 270 (32.7) | 33 (4.0) | 82 (9.9) | 350 (42.4) |
|  | Median (IQR) | 134 (122–148) | 7 (5–10) | 126 (66–168) | 134 (113–160) | 134 (113–148) | 8 (4–25) | 134 (84–191) | 134 (112–156) |
|  | Mean (SD) | 140 (49.2) | 10 (11.9) | 123 (63.6) | 140 (56.8) | 134 (50.5) | 27 (46.5) | 132 (66.6) | 137 (56.4) |
| Enzalutamide | n (%) | . | . | 25 (4.5) | 25 (4.5) | . | 826 (100.0) | 24 (2.9) | 826 (100.0) |
|  | Median (IQR) | . | . | 132 (57–309) | 132 (57–309) | . | 164 (84–296) | 29 (29–114) | 168 (84–308) |
|  | Mean (SD) | . | . | 210 (215.5) | 210 (215.5) | . | 225 (209.0) | 95 (134.2) | 227 (198.1) |
| First-generation anti-androgen therapy^c^ | n (%) | 328 (59.0) | 129 (23.2) | 55 (9.9) | 340 (61.2) | 545 (66.0) | 196 (23.7) | 65 (7.9) | 557 (67.4) |
|  | Median (IQR) | 106 (50–221) | 30 (16–57) | 64 (41–158) | 125 (58–289) | 115 (57–239) | 27 (14–54) | 57 (29–139) | 137 (58–268) |
|  | Mean (SD) | 193 (285.4) | 47 (43.7) | 126 (151.7) | 224 (316.9) | 182 (202.7) | 57 (87.4) | 152 (253.7) | 216 (263.6) |
| Other^d^ | n (%) | 89 (16.0) | . | 306 (55.0) | NC | 106 (12.8) | . | 477 (57.7) | NC |

AA, abiraterone acetate; ADT, androgen-deprivation therapy; ENZA, enzalutamide; IQR, interquartile range; mCRPC, metastatic castration-resistant prostate cancer; NC, not calculated; SD, standard deviation.

A period (.) means that data were not applicable at that time point.

Cisplatin data are not shown because <5 patients received this treatment before, during, or after index in both AA and ENZA treatment groups.

^a^ Corticosteroid-containing regimens included AA, cabazitaxel, and docetaxel.

^b^ The category ‘ADT alone’ differs from the use of ADT pre-index reported at baseline, because ADT alone only included ADT when it was reported as the main treatment component (i.e., ADT use without other reported medications that are commonly co-administered with ADT). Specifically, ADT alone was defined by patients who had:

- ADT treatment before the index date
- ADT treatment after discontinuing treatment with ENZA or AA and did not die
- ADT treatment, and ADT was the main treatment component listed (i.e., did not have any of the other treatments concomitantly)

^c^ First-generation anti-androgen therapy included bicalutamide, flutamide, nilutamide, cyproterone acetate.

^d^ Other includes no treatment, non-categorized treatment, or death

### Online Resource 3.

#### **Treatment duration of corticosteroid-containing PC regimens before, during, and after AA or ENZA index treatment, based on the total population with mCRPC in the EVADE study**

| Treatment/  Duration^a^ (days) | Statistic | AA (n = 556) | | | | ENZA (n = 826) | | | |
| --- | --- | --- | --- | --- | --- | --- | --- | --- | --- |
|  |  | Pre-index | During index | Post-index | Overall | Pre-index | During index | Post-index | Overall |
| Corticosteroid-containing PC regimens^b^ | N | 556 | 556 | 556 | 556 | 826 | 826 | 826 | 826 |
|  | Median (IQR) | 0 (0–134) | 140 (82–281) | 0 (0–0) | 246 (127–394) | 0  (0, 113) | 0 (0–0) | 0  (0–0) | 0 (0–135) |
|  | Mean (SD) | 54 (83.1) | 218 (217.8) | 26 (69.5) | 298 (233.4) | 46 (74.6) | 1 (7.3) | 25 (71.4) | 72 (100.3) |

AA, abiraterone acetate; CV, cardiovascular; CVD, cardiovascular disease; ENZA, enzalutamide; IPTW, inverse probability of treatment weighting; IQR, interquartile range; mCRPC, metastatic castration-resistant prostate cancer.

^a^ The total population was used as the denominator for the calculation of time on treatment before, during, and after index treatment. Treatment duration was assigned a value of zero for patients who did not receive treatment with corticosteroids.

^b^ Corticosteroid-containing regimens included AA, cabazitaxel, and docetaxel.

### Online Resource 4.

#### **Proportion of patients with type 2 diabetes and CV comorbidities recorded in primary and secondary care prior to the index date in patients with mCRPC in the EVADE study**

| Comorbidities | AA (n = 556) | | ENZA (n = 826) | | Total (N = 1382) | |
| --- | --- | --- | --- | --- | --- | --- |
|  | Recorded in primary (%) | Recorded in secondary (%) | Recorded in primary (%) | Recorded in secondary (%) | Recorded in primary (%) | Recorded in secondary (%) |
| T2DM | 91.4 | 56.9 | 96.8 | 61.3 | 94.9 | 59.8 |
| **CV comorbidities** | 82.1 | 90.1 | 85.6 | 85.1 | 84.2 | 87.1 |
| Ischemic heart disease | 54.6 | 96.9 | 53.9 | 97.6 | 54.2 | 97.3 |
| Hypertension | 78.6 | 88.2 | 82.0 | 83.2 | 80.7 | 85.1 |
| Arrhythmia | 13.7 | 94.7 | 15.0 | 89.5 | 14.5 | 91.7 |
| Congestive heart failure | 60.0 | 64.0 | 67.2 | 65.6 | 65.1 | 65.1 |
| Atherosclerosis and embolic events | 41.3 | 58.7 | 51.2 | 58.1 | 47.7 | 58.3 |
| Cerebrovascular disease | 82.0 | 59.0 | 82.8 | 68.8 | 82.4 | 64.0 |

AA, abiraterone acetate; CV, cardiovascular; ENZA, enzalutamide; ICD-10, International Classification of Diseases and Related Health Problems – version 10; mCRPC, metastatic castration-resistant prostate cancer; T2DM, type 2 diabetes mellitus.

Data shown are proportions of patients with comorbidities recorded in primary or secondary care from the total population of patients with comorbidities recorded in either database. Patients may have had a comorbidity recorded in both primary and secondary care.

Recorded in primary: Calculated as the number of patients with the comorbidity recorded in primary care divided by the total number of patients with the comorbidity recorded (i.e., recorded in either primary care or secondary care), multiplied by 100.

Recorded in secondary: Calculated as the number of patients with the comorbidity recorded in secondary care divided by the total number of patients with the comorbidity recorded (i.e., recorded in either primary care or secondary care), multiplied by 100.

For this description of characteristics recorded in either primary or secondary care data, the ICD-10 codes were diabetes (ICD-10 codes E10, E11, E12, E13, E14, E88.8); hypertension (ICD-10 code I10); ischemic heart disease (ICD-10 codes I20, I21, I24, I46, I22, I23, I25); arrhythmia (ICD-10 codes I47, I48, I49); congestive heart failure (ICD-10 codes I42, I50); cerebrovascular disease (ICD-10 codes G45, I60, I61, I62, I63, I64, I65, I66, I67, I69, I69); atherosclerosis and embolic events (ICD-10 codes I70, I71, I72, I73, I74, I77, I79).

#### **Proportion of comorbidities recorded in primary and secondary care of those recorded in either primary or secondary care**


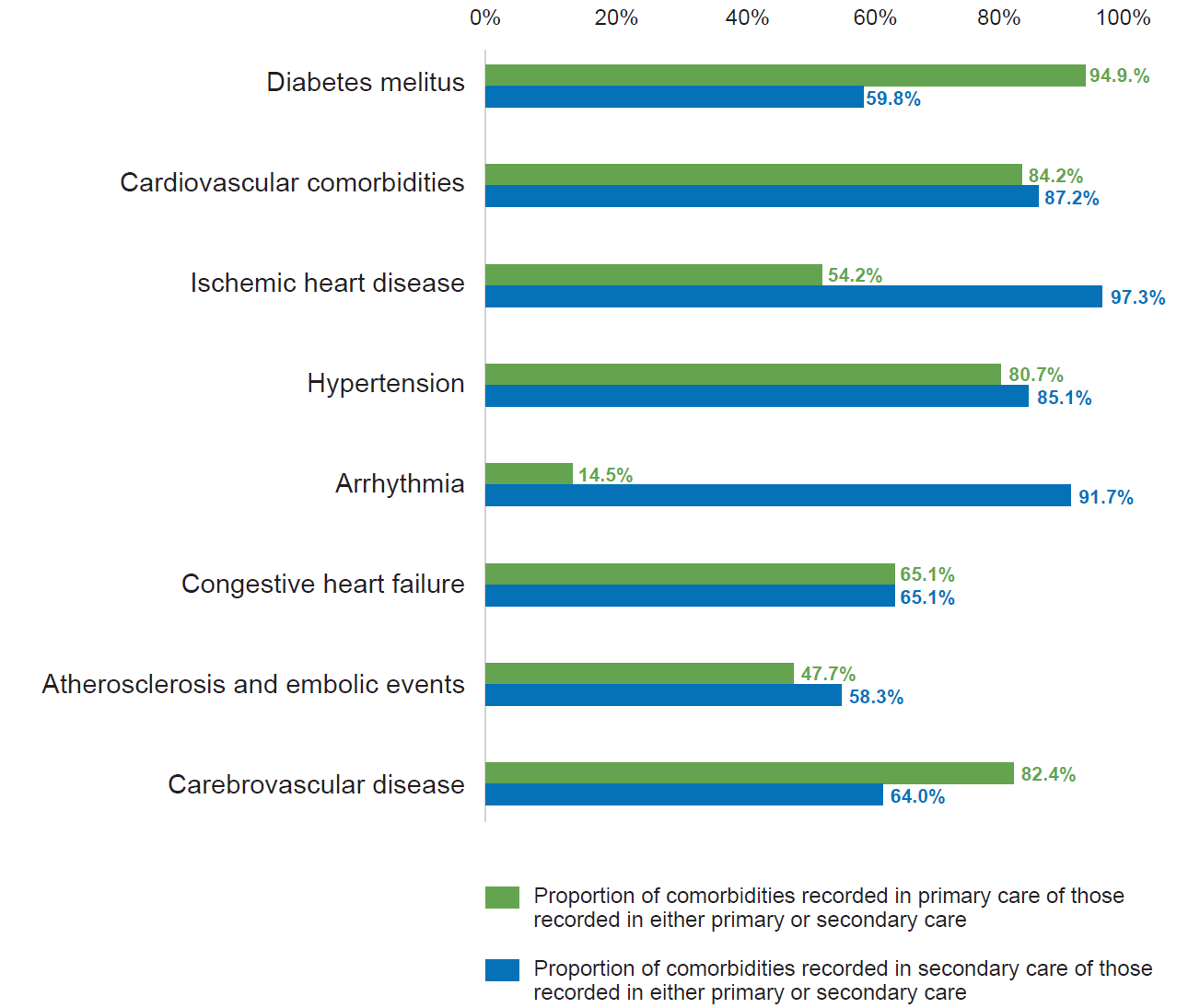


Data shown are proportions of patients with comorbidities recorded in primary or secondary care from the total population of patients with comorbidities recorded in either database. Patients may have had a comorbidity recorded in both primary and secondary care.

For secondary care data, the ICD-10 codes for comorbidities in the PROCCO study were used.
